# Nanogenerator-Based Self-Charging Energy Storage Devices

**DOI:** 10.1007/s40820-019-0251-7

**Published:** 2019-03-09

**Authors:** Kun Zhao, Yuanhao Wang, Lu Han, Yongfei Wang, Xudong Luo, Zhiqiang Zhang, Ya Yang

**Affiliations:** 1 CAS Center for Excellence in Nanoscience, Beijing Key Laboratory of Micro-Nano Energy and Sensor, Beijing Institute of Nanoenergy and Nanosystems, Chinese Academy of Sciences, Beijing, 100083 People’s Republic of China; 2 State Key Laboratory of Advanced Processing and Recycling of Nonferrous Metals, School of Materials Science and Engineering, Lanzhou University of Technology, Lanzhou, 730050 People’s Republic of China; 3 Xinjiang Technical Institute of Physics and Chemistry, Chinese Academy of Sciences, Ürümqi, 830011 Xinjiang People’s Republic of China; 4 School of High Temperature Materials and Magnesite Resources Engineering, University of Science and Technology Liaoning, 185 Qianshan Zhong Road, Anshan, 114044 People’s Republic of China; 5 School of Chemical Engineering, University of Science and Technology Liaoning, 185 Qianshan Zhong Road, Anshan, 114044 People’s Republic of China; 6 School of Nanoscience and Technology, University of Chinese Academy of Sciences, Beijing, 100049 People’s Republic of China

**Keywords:** Nanomaterial, Nanogenerator, Energy storage device, Self-charging

## Abstract

One significant challenge for electronic devices is that the energy storage devices are unable to provide sufficient energy for continuous and long-time operation, leading to frequent recharging or inconvenient battery replacement. To satisfy the needs of next-generation electronic devices for sustainable working, conspicuous progress has been achieved regarding the development for nanogenerator-based self-charging energy storage devices. Herein, the development of the self-charging energy storage devices is summarized. Focus will be on preparation of nanomaterials for Li-ion batteries and supercapacitors, structural design of the nanogenerator-based self-charging energy storage devices, performance testing, and potential applications. Moreover, the challenges and perspectives regarding self-charging energy storage devices are also discussed.

## Highlights

- The progress of nanogenerator-based self-charging energy storage devices is summarized.
- The fabrication technologies of nanomaterials, device designs, working principles, self-charging performances, and the potential application fields of self-charging storage devices are presented and discussed.
- Some perspectives and problems that need to be solved are described.

## Graphical abstract

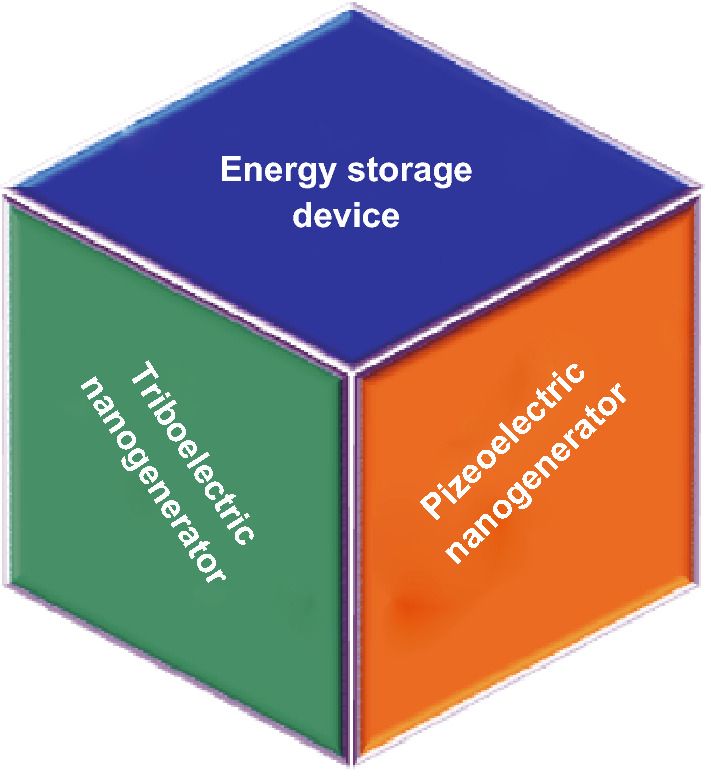

## Introduction

With the rapid development of economy and society, microelectronic devices are playing an increasingly important role in our daily lives. Usually, these devices can be powered using lithium-ion batteries or supercapacitors, which require external power sources to periodically charge them due to their limited capacities [1–3]. Moreover, it will cost a significant quantity of manpower, financial resources, and time, especially in remote areas. For power supply, scientists are researching new methods of scavenging clean energies from the surrounding environment [6–10]. Based on this background, the piezoelectric and triboelectric nanogenerators were invented by Wang et al. in 2006 and 2012, respectively [4, 5], which can effectively convert small mechanical energy into electrical energy in the ambient environment, such as wind energy [6, 7], wave energy [8], droplet energy [9], and other mechanical energies [10]. They are clean or wasted energies in our surrounding environment. Nanogenerators not only can effectively scavenge mechanical energies mentioned above, but also have several advantages such as simple, small, light, low cost, no auxiliaries, and convenient. They can be applied to wireless sensors and microelectronics devices. Currently, research and development of self-powered electronic devices have become a hot topic among scientists [11–28]. In particular, remarkable progress has been made in the field of self-charging power textile for wearable electronics [29–33]. Thus, it is important to investigate self-charging energy storage devices that can effectively integrate energy harvesting and storage units in one device for powering some small electronic devices with sustainable energy supply.

This review focuses on the progress of nanogenerator-based self-charging energy storage devices in recent years. The fabrication technologies of nanomaterials, device designs, working principles, self-charging performances, and the potential application fields of self-charging storage devices are presented and discussed here. Moreover, some perspectives and problems that need to be solved are also described, which can pave the path for practical applications.

## Nanomaterials

Due to the large specific surface area and excellent energy storage characteristics, nanomaterials demonstrate a reversible capacity higher than that of the commercial products. New nanomaterials have fundamental advancements regarding energy storage and conversion devices, both of which are important to satisfy the challenges of the finite nature of fossil fuels and environmental problems. Over these years, scientists have conducted a wide range of research and achieved a series of experimental progresses.

**Fig. 1:**
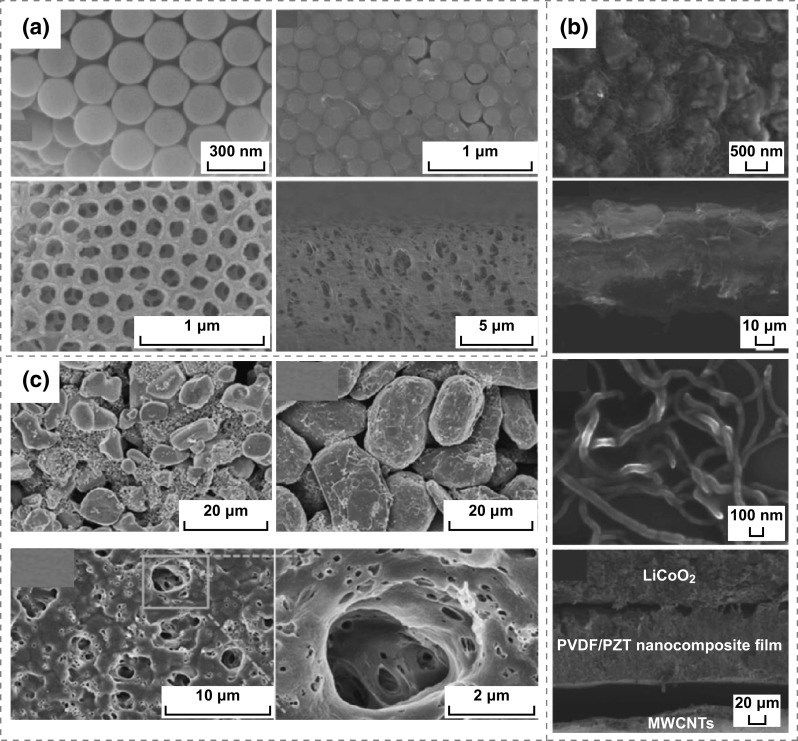
**a** SEM images of SiO_2_ microspheres, SiO_2_-PVDF composite film, mesoporous PVDF film after removing SiO_2_ microspheres on the top view, and the mesoporous PVDF film on the side view. Reprinted with permission from Ref. [11]. **b** FESEM images of the PVDF–PZT nanocomposite film, FESEM image of the PVDF–PZT nanocomposite film, FESEM image of MWCNTs, and cross-sectional FESEM image of the SCPC device (LiCoO_2_ as cathode, PVDF–PZT nanocomposite film as piezo-separator and MWCNTs as anode). Reprinted with permission from Ref. [12]. **c** Morphological images of the cathode (LiCoO_2_), anode (artificial graphite), and PVDF piezo-separator. Reprinted with permission from Ref. [13]

**Fig. 2:**
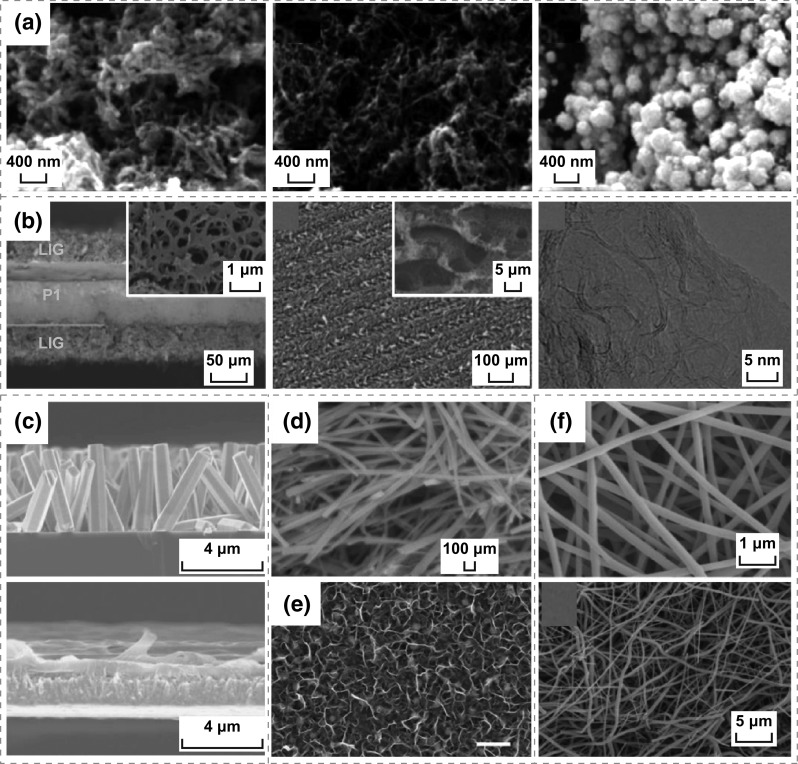
**a** SEM images of PANI/CNT, unmodified or PtNP-modified PPy/CNT surfaces. Reprinted with permission from Ref. [14]. **b** Cross-sectional SEM image of a double-sided laser-engraved PI substrate with both sides of LIG, SEM image of the LIG thin film, and HRTEM image obtained from the edge of a LIG flake. Reprinted with permission from Ref. [15]. **c** Cross-sectional SEM images of ZnO NW arrays and PVDF mesoporous nanostructured film. Reprinted with permission from Ref. [16]. **d** FESEM image of MnO_2_ nanostructure. Reprinted with permission from Ref. [17]. **e** SEM images of the 3D Au@MnO_2_ nanocomposites (scale bar, 500 nm). Reprinted with permission from Ref. [18]. **f** SEM images of the LiMn_2_O_4_ precursor NWs and the obtained carbon NWs. Reprinted with permission from Ref. [19]

**Fig. 3:**
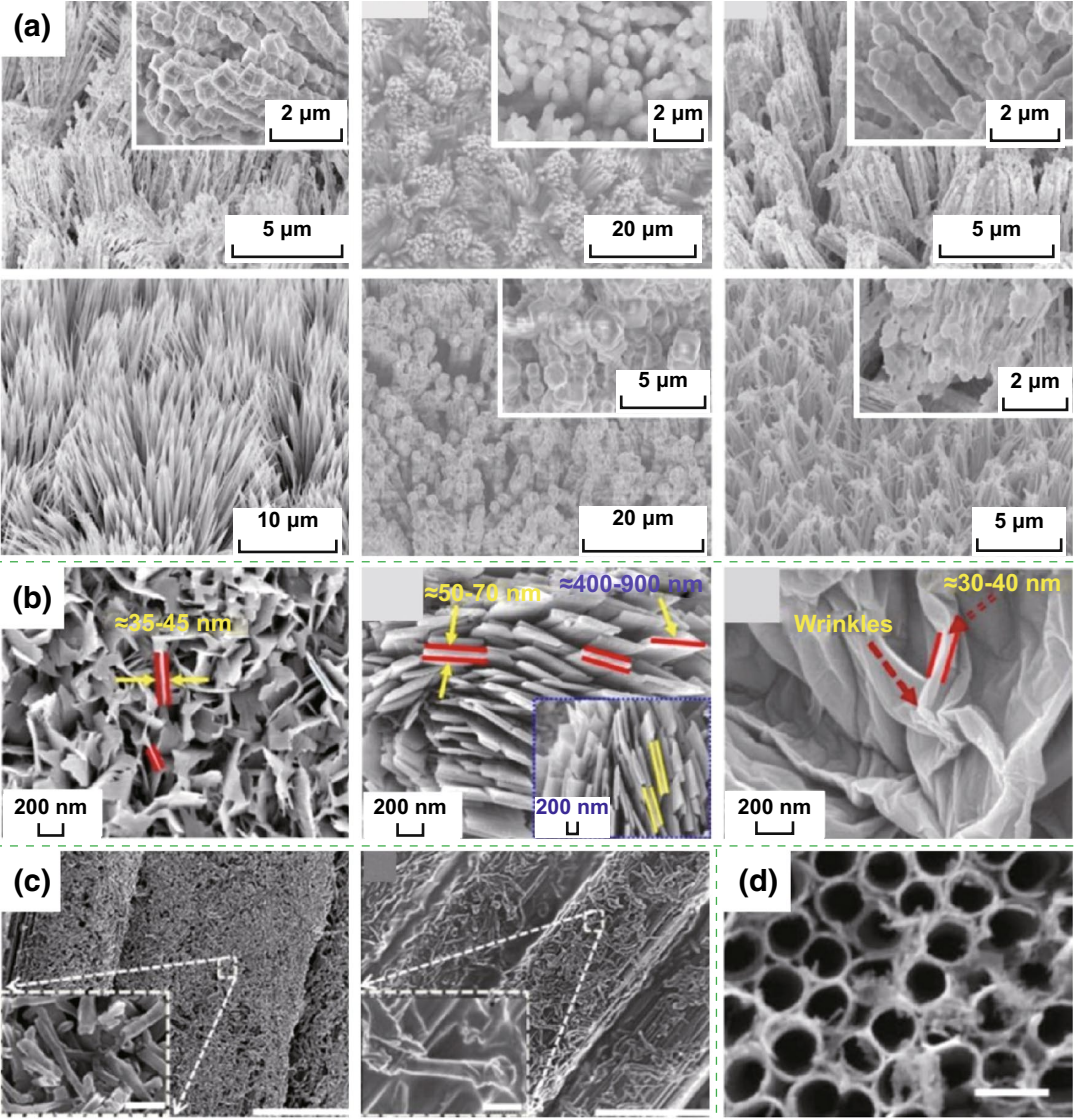
**a** SEM images of ZnCo_2_O_4_@Zn-Co-S HA, as-prepared CNTFs@Zn/Co-Zif, ZnCo_2_O_4_@ZnCo_2_O_4_ HA, CNTFs@H–Co_3_O_4_ NA, CNTFs@H–Co_3_O_4_@ZIF-67 HA, and CNTFs@H–Co_3_O_4_@CoNC HA. Reprinted with permission from Ref. [20]. **b** FESEM images of CuO nanoflake arrays grown on Cu foil, hierarchical NiCoOH nanoplies electrodeposited on CuO@Cu foil and RGO nanosheets. Reprinted with permission from Ref. [21]. **c** SEM images of a CNF-coated yarn and a PEDOT: PSS/CNF-coated yarn (scale bar, 10 μm). Reprinted with permission from Ref. [22]. **d** SEM images of the TiO_2_ nanotube arrays on the Ti wire (scale bar, 100 nm). Reprinted with permission from Ref. [23]

Nanomaterials can not only be used as positive and negative electrode materials, but also be used as special electrolytes or separators. In 2017, He et al. fabricated a novel all-solid-state self-charging power cell (SCPC) using mesoporous polyvinylidene difluoride (PVDF)–LiPF_6_ film as piezoelectrolyte [11]. The morphology and microstructure of SiO_2_ and PVDF film are presented in Fig. 1a, demonstrating that the average diameters of SiO_2_ are approximately 200 nm, and the pore size and thickness of PVDF film prepared using SiO_2_ as template are 200 nm and 1 μm, respectively. Figure 1b presents that a PVDF–PZT (Lead zirconate titanate) nanocomposite film and multi-walled carbon nanotubes (MWCNTs) have been used as a piezo-separator and negative material in SCPC [12]. It can be distinctly observed that the thickness of the PVDF–PZT nanocomposite film is approximately 60–110 μm; the MWCNTs have lengths of 5–15 µm and diameters of 40–60 nm, respectively. Kim et al. designed a SCPC consisting of LiCoO_2_ nanomaterials as the cathode and artificial graphite as the anode with a porous ZnO-free PVDF separator [13], as illustrated in Fig. 1c. Figure 2a displays that the nano-polyaniline/carbon nanotube (PANI/CNT) and nano-polypyrrole (PPy)/CNT were utilized to fabricate a hybrid electric power device (HEPD) [14]. The porous multilayer structures of laser-induced graphene (LIG) electrodes were used to fabricate the TENG and micro-supercapacitor, as depicted in Fig. 2b [15]. As illustrated in Fig. 2c, ZnO nanowire (NW) arrays and PVDF mesoporous nanostructured films have been used in a piezo-driven SCPC [16]. Ramadoss et al. fabricated a piezoelectric effect-driven self-charging supercapacitor power cell (SCSPC) using MnO_2_ NWs as positive and negative electrodes (Fig. 2d) and a PVDF–ZnO film as a separator [17]. Luo et al. successfully applied 3D Au@MnO_2_ nanocomposites into the transparent and flexible self-charging power film (SCPF), as exhibited in Fig. 2e [18]. Figure 2f displays the morphology of LiMn_2_O_4_ precursor NWs and carbon NWs by electrospinning and annealing calcinations, where the LiMn_2_O_4_ precursor NWs exhibit smooth surfaces with diameters ranging from 150 to 260 nm and lengths of approximately several tens of micrometers. The polyvinylpyrrolidone NWs were oxidized under air atmosphere and then were carbonized under Ar protection at 850 °C to obtain conductive carbon NWs as the anode of Li-ion batteries (LIBs) [19]. Zhao et al. developed a new strategy for fabricating a metal–organic framework (MOF) for the template-directed growth of hierarchically well-oriented NW arrays based on carbon nanotube fibers (CNTFs) for electrochemical supercapacitors, where the corresponding SEM images of the MOFs, MOF derivatives, and CNTFs materials are illustrated in Fig. 3a [20]. Researchers have prepared CuO nanoflake arrays, hierarchical NiCoOH nanoplies and reduced graphene oxide (RGO) nanosheets for energy storage devices. As displayed in Fig. 3b, a uniform and homogeneous growth of CuO nanoflake arrays with an average thickens of 35–45 nm on Cu foil substrate and the 3D architectures of NiCoOH nanoplies with an average wall thickness of 50–70 nm were successfully deposited on CuO@Cu foil, and the wrinkled sheets of RGO with an average thickness of 30–40 nm were evident [21]. The all-solid-state symmetric yarn SC was fabricated using dip-coating carbon nanofiber (CNF) and poly(3,4-ethylenedioxythiophene)-poly-(styrenesulfonate) (PEDOT: PSS) on a carbon fiber (CF) bundle, as depicted in Fig. 3c [22]. Wen et al. used highly ordered TiO_2_ nanotube arrays in F-DSSC, as illustrated in Fig. 3d [23]. The SCPC was fabricated using PVDF/0.5(Ba_0.7_Ca_0.3_) TiO_3_–0.5Ba(Zr_0.2_Ti_0.8_)O_3_ (BCT–BZT) nanocomposite as piezo-separator [36]. Two-dimensional materials have excellent properties, such as open ion diffusion channels and abundant active surfaces that enable fast transport and storage ions. Therefore, it is very important for self-charging energy storage devices. Pazhamalai et al. fabricated the SCSPC using 2D-MoSe_2_ nanosheets as the electrodes, PVDF-co-HFP/TEABF_4_-based ionogel as the electrolyte, and the electrospun PVDF/NaNbO_3_ nanofibrous mat as piezo-separator [37].

## Li-ion Batteries and Supercapacitors

**Fig. 4:**
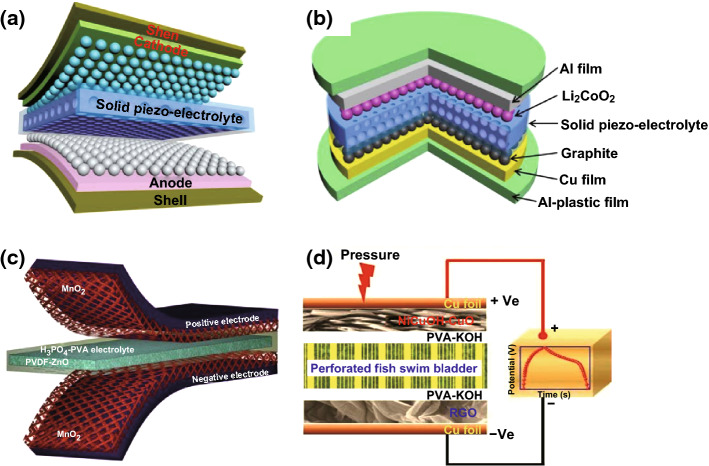
Schematic diagram showing the design of all-solid-state SCPCs (**a**) and the design of a flexible SCPC (**b**). Al-plastic film is used as the shells of flexible SCPCs. Reprinted with permission from Ref. [11]. **c** Schematic diagram of the fabricated SCSPC. MnO_2_ on aluminum foil is used as the positive and negative electrodes and PVDF–ZnO film as a separator. Reprinted with permission from Ref. [17]. **d** The effect of mechanical deformation (by pressure) on the SCASC device. Reprinted with permission from Ref. [21]

**Fig. 5:**
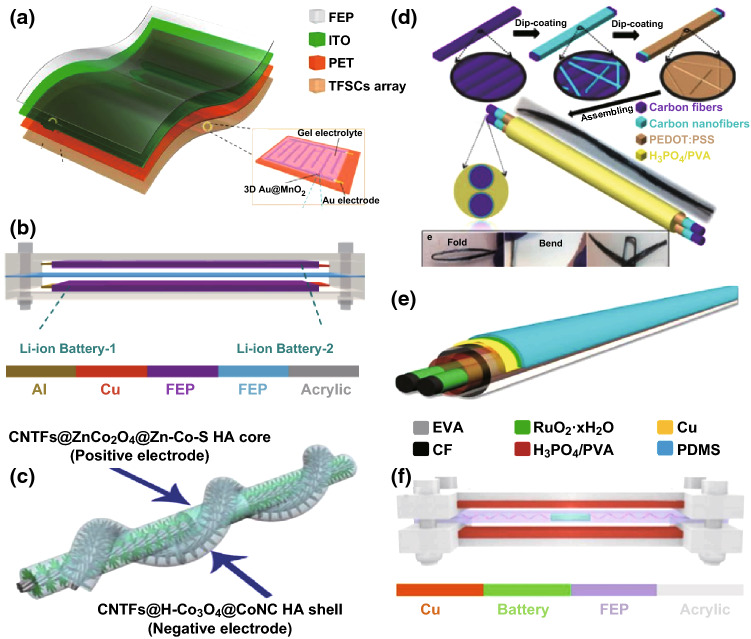
Schematic illustration of **a** the transparent and flexible SCPF. Reprinted with permission from Ref. [18]. **b** Self-charging Li-ion batteries. Reprinted with permission from Ref. [19]. **c** the assembled structure of the FASC. Reprinted with permission from Ref. [20]. **d** Fabrication process of the all-solid-state symmetric yarn SC composed of two PEDOT:PSS/CNF/CF electrodes in parallel. Reprinted with permission from Ref. [22]. Schematic diagram of **e** a single F-SC, consisting of two carbon fibers coated with RuO_2_·xH_2_O in the H_3_PO_4_/PVA electrolyte. Reprinted with permission from Ref. [23]. **f** the fabricated device. Reprinted with permission from Ref. [25]

**Fig. 6:**
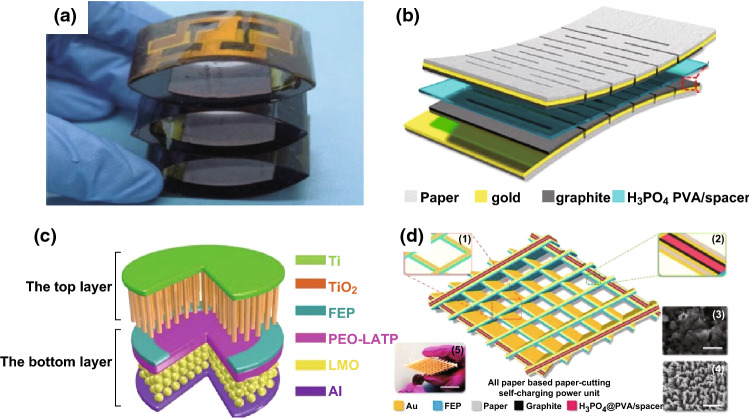
**a** Photograph of an as-fabricated self-charging power system, including three EP-TENGs in parallel and three EP-SCs in series (scale bar, 1 cm). Reprinted with permission from Ref. [24]. **b** Schematic structure of a kirigami-based supercapacitor (KP-SC). Reprinted with permission from Ref. [26]. **c** Fabrication of the convoluted power device. Reprinted with permission from Ref. [27]. **d** Structural scheme of the PC-SCPU. Insets 1 and 2 show the basic working component of the P-TENG and P-SC. Insets 3 and 4 are the SEM image of the graphite-based electrode (scale bar, 2 μm) for the P-SC and the nanostructured FEP film (scale bar, 2 μm) for the P-TENG. Inset 5 shows a photograph of the as-prepared P-SCPU, which is compared with a coin (scale bar, 3 cm). Reprinted with permission from Ref. [28]

Currently, LIBs and supercapacitors are widely utilized as the main electrochemical energy storage devices. They can be used as the energy supply units for powering mobile phones, personal wearable devices, microelectronic devices, etc. The reported self-charging energy storage devices are mainly based on LIBs and supercapacitors. These devices can collect and convert mechanical energy into electric energy in the surrounding environment, and then store the scavenged energy as chemical energy. Energy scavenging function of the devices can be realized by piezoelectric nanogenerators or triboelectric nanogenerators. Figure 4a–d depicts the self-charging energy storage devices based on the piezoelectric effect [11, 17, 21]. The mesoporous PVDF–LiPF_4_ film (Fig. 4a, b) and PVDF–ZnO film (Fig. 4c) are used in the device, where a PVA–KOH gel electrolyte-soaked perforated fish swim bladder (Fig. 4d) was used as piezoelectric separator. Meanwhile, the flexible self-charging energy storage devices using piezoelectric nanogenerator have been developed. Yuan et al. reported a paper-based flexible SC using PANI/Au/paper as electrodes, which can be charged by a piezoelectric generator [38]. The voltage of six SCs connected in series can be charged to 2.6 V in approximately 11 h; they could power a blue LED for 5 min. As presented in Figs. 5a–f and 6a–d, the LIBs and supercapacitors were based on triboelectric nanogenerators [18–28]. Figure 5a displays the schematic diagrams of all-solid-state transparent and flexible supercapacitors (TFSCs) based on interdigital electrodes of 3D Au@MnO_2_ nanocomposites, which were connected to form an array on the backside of the nanogenerator [18]. The flexible LIBs based on electrospun LiMn_2_O_4_ NWs as cathode and carbon NWs as anode are presented Fig. 5b [19]. Figure 5c displays the schematic diagram of flexible fiber-shaped coaxial asymmetric supercapacitors, where it was assembled by twisting together the CNTFs@ZnCo_2_O_4_@Zn–Co–S positive electrode (core) and the CNTFs@H–Co_3_O_4_@CoNC HA negative electrode [20]. Dong et al. fabricated the all-solid-state symmetric yarn supercapacitor (SC) using dip-coating carbon nanofiber (CNF) and poly(3,4-ethylenedioxythiophene)-poly-(styrenesulfonate) (PEDOT:PSS) on a carbon fiber (CF) bundle, as presented in Fig. 5d [22]. Figure 5e illustrates that the fiber-shaped SCs (F-SCs) were fabricated using RuO_2_·xH_2_O-coated carbon fibers and PVA/H_3_PO_4_ gel electrolyte [23]. Gao et al. reported a novel strategy of implanting a solid Li-ion battery (SLB) into a triboelectric fluorinated ethylene propylene (FEP) film of TENG to simultaneously scavenge and store wind energy as chemical energy, where the SLB was prepared using the counter rolling mechanism in the order of the anode, PEO–LATP membrane, and cathode, as displayed in Fig. 5f [25]. Figure 6a presents that three EP-TENGs connected in parallel were integrated with three EP-SCs connected in series to create a self-charging power system, where the conductive carbon paper acted as capacitive materials, while the PAN paper served as separator [24]. The flexible paper-based supercapacitor is displayed in Fig. 6b, where the graphite, H_3_PO_4_/PVA, and kirigami were utilized as the active material, electrolyte, and separator, respectively [26]. Liu et al. reported a convoluted power device by internally hybridizing a TENG and an SLB by sharing common electrodes. The SLB consists of TiO_2_ nanotubes as anode, the polyethylene oxide-Li_(1+x)_Ti_(2−x)_Al_x_(PO4)_3_ (PEO–LATP) as solid electrolyte, and the LiMn_2_O_4_ nanoparticles as cathode (Fig. 6c) [27]. Figure 6d presents the schematic diagram of the all-paper-based cut-paper self-charging power unit (PC-SCPU), where it can be distinctly observed that the supercapacitor is composed of graphite as active material and H_3_PO_4_/PVA as electrolyte [28].

## Self-Charging Principles

**Fig. 7:**
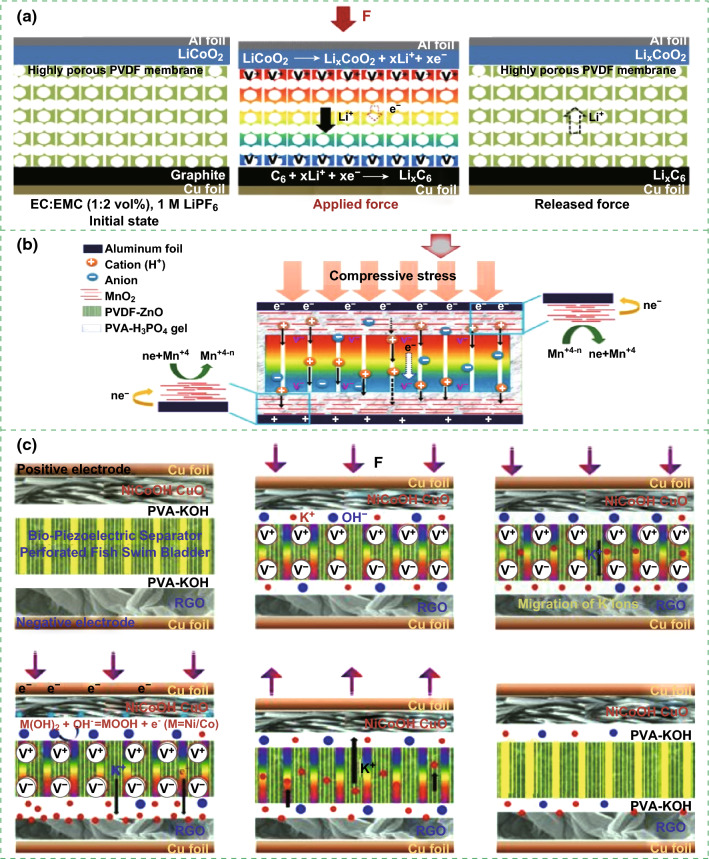
**a** Schematic self-charging mechanism induced by a porous PVDF piezo-separator in Li-ion secondary batteries. Reprinted with permission from Ref. [13]. **b** Working mechanism of the SCSPC driven by mechanical deformation. Reprinted with permission from Ref. [17]. **c** Typical working mechanism of the solid-state SCASC under applied mechanical deformations. Reprinted with permission from Ref. [21]

There are two typical charging principles of self-charging energy storage devices. One is based on piezoelectric potential-driven electrochemical oxidation and reduction reaction. For piezoelectric nanogenerator-based self-charging lithium-ion batteries, the piezoelectric field created by the PVDF piezo-separator forms along the z-axis from the cathode side of the system when compressive force is applied to the battery surface. The piezoelectric potential of the porous PVDF separator causes Li^+^ ions to migrate from the cathode to the anode through ion-conducting separator and electrolyte, resulting in the incorporation of Li^+^ ions into the anode electrode. An electron can take the same pathway to maintain charge neutrality, where this migration represents complete conversion of mechanical energy into electrochemical energy, as displayed in Fig. 7a [13]. For piezoelectric nanogenerator-based self-charging supercapacitors, since no pressure force is applied to the device, there is no electrochemical reaction due to the electrochemical equilibrium between the electrodes and electrolyte. A compressive stress can be caused by the polarization of the separator (PVDF–ZnO or BPES, Fig. 7b, c) due to the piezoelectric effect. Moreover, the separator produces a potential difference along the thickness direction. These positive and negative piezoelectric potentials were generated at the top and the bottom of the film, driving the electrolytic ions to move toward the positive and negative electrodes. The piezoelectric field induced cationic movement in the electrolyte to screen the generated piezopotential across the separator. This ionic movement induces an electrochemical imbalance in the electrolyte and the positive/negative electrodes. As a result, the positive and negative potentials were developed on the upper and lower surface of the separator that can drive the electrolyte ions toward the different electrodes. The charging process can be continued to accomplish equilibrium of the two electrodes with the generated piezoelectric potentials. The devices directly convert the mechanical energy into electrochemical energy in the SCs, as presented in Fig. 7b, c [17, 21], respectively.

**Fig. 8:**
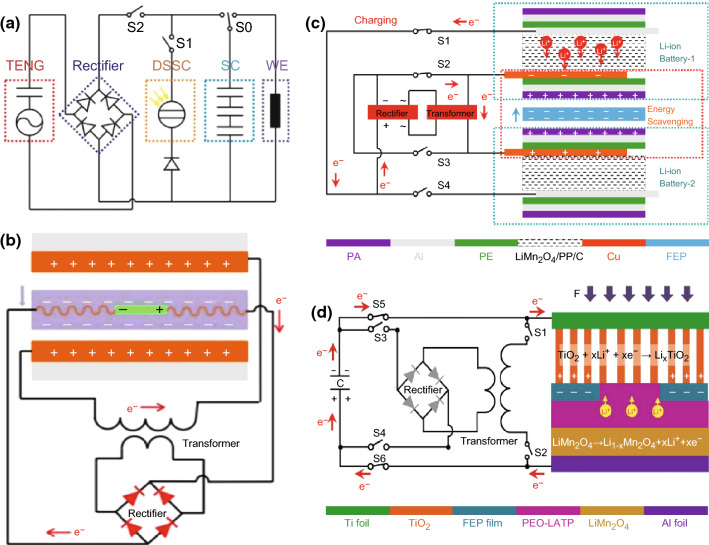
**a** Circuit diagram of the self-charging powered textile for wearable electronics (WE). Reprinted with permission from Ref. [23]. **b** Implanting an SLB into a triboelectric nanogenerator. Reprinted with permission from Ref. [25]. **c** Schematic diagram of self-charging process for the Li-ion batteries, distinctly showing the flow of electrons in the circuit. Reprinted with permission from Ref. [19]. **d** Schematic illustration of the electric storage process from the external capacitor to the convoluted power device as an SLB. Reprinted with permission from Ref. [27]

Another charging principle is based on triboelectrifcation. Firstly, a TENG can be utilized to convert mechanical energy into electric energy, and then a bridge rectifier is used to convert the generated AC current of TENG into direct current signals before charging the energy storage devices, as displayed in Fig. 8a [23]. The electrical outputs generated by TENGs have the characteristics of high voltage and low current signals. Usually, a transformer is used to decrease the voltage and increase the current before rectifying, as displayed in Fig. 8b–d [19, 25, 27] or by adding a power management circuit between the TENGs and energy storage devices to reduce the voltage, increase the current, and turn the AC into DC signals [34]. More importantly, Fig. 8c, d displays the self-charging process of the LIBs that can simultaneously generate and store electric energy by itself, which is an important step toward next-generation LIBs for pushing the practical applications in self-powered electronic devices.

Pu et al. reported a method to solve the problem of the impedance match between the TENG and a battery with appropriate design of transformers [35]. It provides an effective method to solve the problem of impedance match between TENGs and energy storage devices.

## Self-Charging Performances

**Fig. 9:**
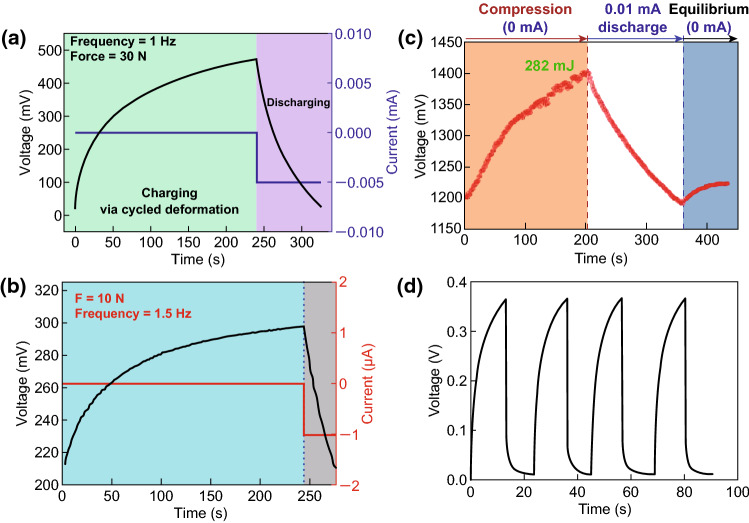
**a** Self-charging process of an all-solid-state SCPC under periodic applied force. Reprinted with permission from Ref. [11]. **b** Self-charging process applying cycled mechanical compressive strain to the device based on PVDF–PZT nanocomposite film as piezo-separator. Reprinted with permission from Ref. [12]. **c** A typical self-charging profile comprising three regions: a compression region inducing self-charging over an initial 200 s when continuous mechanical compression of 282 mJ was applied at 1 Hz to the cell. Reprinted with permission from Ref. [13]. **d** The device was charged at a constant current of 5 mA and discharged at a constant load of 5 kΩ in an N_2_ saturated buffer. Reprinted with permission from Ref. [14]

**Fig. 10:**
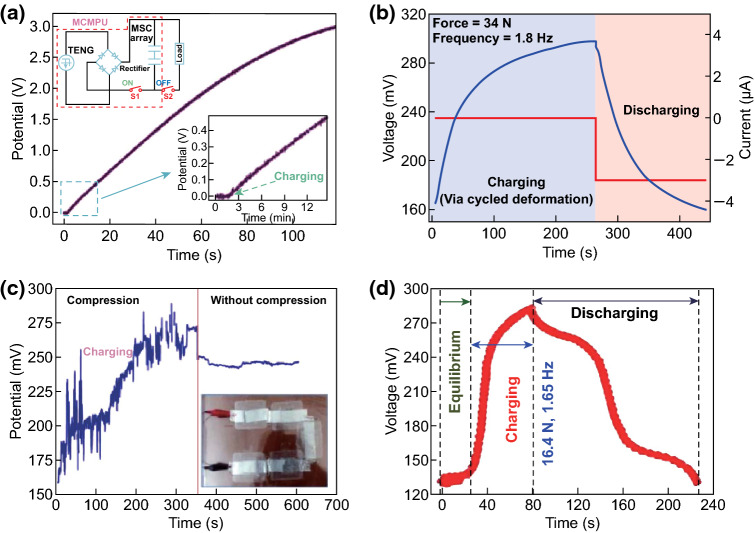
**a** Charging curve of the MSC array component. Reprinted with permission from Ref. [15]. **b** A typical self-charging process under periodic compressive stress of a SCPC fabricated using PVDF mesoporous nanostructured film as piezo-separator. Reprinted with permission from Ref. [16]. **c** Self-charging process of the serially connected five SCSPCs under periodic compressive strain provided by human palm impact to the whole devices. Reprinted with permission from Ref. [17]. **d** The self-charging-discharging profile under continuous human finger imparting. Reprinted with permission from Ref. [21]

**Fig. 11:**
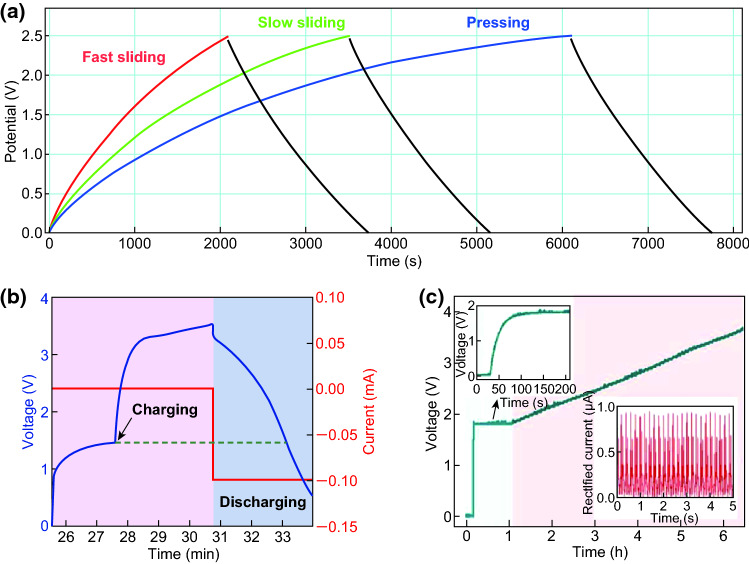
**a** Charging curves of four TFSCs in series charged by the TF-TENG at fast sliding, slow sliding, and pressing. Reprinted with permission from Ref. [18]. **b** Self-charging and discharging curves of the top Li-ion battery. Reprinted with permission from Ref. [19]. **c** Charging curve of the F-DSSC and the F-TENG, where the light blue-shaded area corresponds to the charging curve of the F-DSSC and the light red-shaded area corresponds to the charging curve of the F-DSSC–F-TENG hybrid. Reprinted with permission from Ref. [23]

**Fig. 12:**
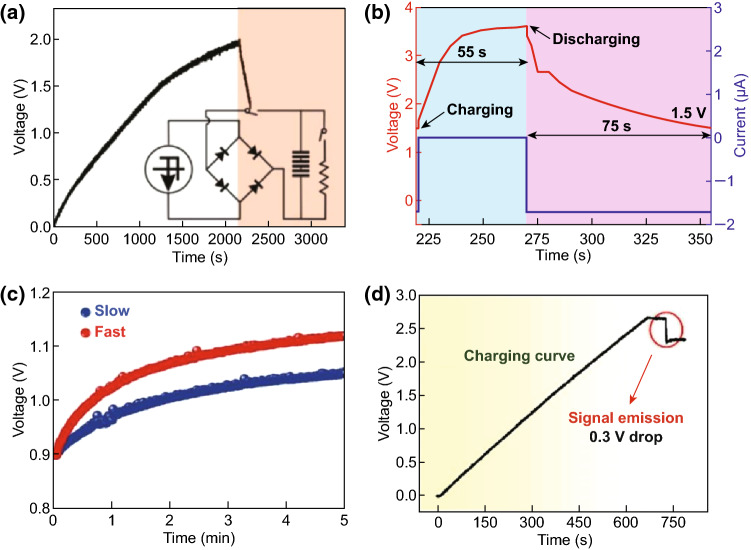
**a** *V*–*t* curve of the 3-series EP-SCs charged by pressing 3-parallel EP-TENGs. Reprinted with permission from Ref. [24]. **b** Charging and discharging curves using the TENG at wind speed of 24.6 m s^−1^. Reprinted with permission from Ref. [25]. **c** Charging curves of another convoluted power device as an SLB using the produced electric energy by human walking. Reprinted with permission from Ref. [27]. **d** *V*–*t* curve of the PC-SCPU under charging mode and then instantaneously driving a wireless remote control. Reprinted with permission from Ref. [28]

The mechanical energy scavenged from environment using nanogenerators can be converted into electricity, which can be then stored in the energy storage devices such as lithium-ion batteries or capacitors using a management circuit. In this process, energy conversion efficiency and self-charging performance are undoubtedly critical parameters. Figure 9a presents the self-charging curves of the all-solid-state SCPC under periodically applied compressive deformations. When a compressive force of 30 N was applied onto the SCPC at the frequency of 1 Hz, the voltage of the SCPC increased from 25 to 473 mV within 240 s, which can be discharged back to its original voltage in 85 s under a constant current of 5 μA. The obtained corresponding capacity of the SCPC is approximately 0.118 μAh [11]. With a compressive force of 10 N applied to the SCPC based on the PVDF–PZT nanocomposite film at a frequency of 1.5 Hz, the voltage of the device can be increased from 210 to 297.6 mV in 240 s. The device was discharged back to its original voltage in 37 s under a constant discharge current of 1 µA, as depicted in Fig. 9b [12]. Figure 9c presents the voltage change curves of the coin cell in the self-charging and discharging processes. When a compression mechanical energy of 282 mJ was applied on the cell at a frequency of 1 Hz, the voltage of the device can be increased from 1.2 to 1.4 V in approximately 200 s. After achieving the self-charging process, the cell was discharged at a constant current of 0.01 mA, resulting in the decrease in voltage to 1.2 V, where the corresponding discharge capacity was 0.4 μAh [13]. Figure 9d displays the charging/discharging curves of HEPD as a SC with a specific capacitance of approximately 0.5 F/cm^2^, when the fuel (ascorbate) and oxidant (O_2_) were absent in the anolyte and catholyte, respectively [14]. The device was externally charged and then discharged, realizing excellent reproducibility for several cycles. The charging curves of the MSC array component are displayed in Fig. 10a, where the stored charge can be increased steadily with increased charging time, and the potential can be approximately 3 V in 117 min [15]. Figure 10b illustrates that the voltage of the SCPC can be increased from 160 to 299 mV in 250 s when the device is under a compressive force of 34 N with a working frequency of 1.8 Hz. After the self-charging process, the device was discharged with a constant current back to its original voltage in 207 s under a discharge current of 3 μA [16]. The self-charging capability of the five SCSPCs in series was tested under the continuous human palm contact to all devices, as displayed in Fig. 10c. Under continuous self-charging process, the open-circuit voltage can be increased from 160 to 280 mV (120 mV charged) in 350 s and is then sustained in 250 s, even after removing the deformation [17]. The self-charging and discharging process of the fabricated device under continuous human finger imparting (at *F* ≈ 16.4 N, *f* = 1.65 Hz) for 80 s is illustrated in Fig. 10d. Although an initial voltage of approximately 130.1 mV was perceived, the device can be charged up to 281.3 mV from its initial voltage after repeated finger imparting. As a result, the device exhibits a distinct voltage increase of 151.2 mV. After removing finger imparting, the device was discharged back to its initial voltage in 145.5 s under a constant discharge current of 10.5 µA [21]. More importantly, Pazhamalai et al. reported that the MoSe_2_ SCSPC device can be charged from 85 to 708 mV under an applied compressive force of 30 N within 100 s [37]. By comparison, it is observed that the MoSe_2_ SCSPC device has better self-charging performance than the devices depicted in Figs. 9a and 10b. The excellent self-charging performance of the fabricated MoSe_2_ SCSPC devices might be related to intercalative type 2D MoSe_2_ energy storing electrode. As displayed in Fig. 11a, the SCPF was charged using different types of finger motions. For finger tapping, the SCPF was charged from 0 to 2.5 V within 6102 s (blue line) and discharged at 1 μA for 1639 s. When the finger motions are slow sliding (0.5 m s^−1^) and fast sliding (0.8 m s^−1^), the charging times can be shortened to 3518 s (green line) and 2094 s (red line), respectively [18]. The following corresponding discharging at 1 μA can still be sustained in approximately 1630 s. Figure 11b presents the self-charging performance of the top Li-ion battery; it can be easily charged from 1.5 to 3.5 V in approximately 3 min under the vibrations of the FEP film induced by a wind blowing at a speed of approximately 10 m s^−1^ though the device [19]. Figure 11c displays the voltage change of the F-DSSC, where it can be charged from 1.8 to 3.5 V in approximately 33 s using F-TENG [23]. Figure 12a depicts the charging curves of the self-charging power system under the stable and fixed frequency of 5 Hz for the practical application. The circuit schematic diagram of the self-charging system is displayed in the inset of Fig. 4l. It requires approximately 2150 s to charge the EP-SCs to 2 V [24]. The SLB can be easily charged from 1.5 to 3.6 V in approximately 55 s under the vibrations of the FEP film induced by a wind blowing with the speed of approximately 24.6 m s^−1^ though the device, as presented in Fig. 12b [25]. The convoluted power device in the human shoe under the fast walking condition can charge a Li-ion battery to a higher voltage of approximately 1.12 V in approximately 5 min as compared with that (1.05 V) under the slow walking condition, as displayed in Fig. 12c [27]. Figure 12d presents the voltage–time curve of a PC-SCPU when charging and instantaneously working (charging frequency: 3 Hz), where it requires charging for approximately 60 s for wireless remote operation [28]. The above research results indicate that the nanogenerator-based self-charging storage devices have good self-charging performances, which can push the practical applications of self-charging devices.

## Applications

**Fig. 13:**
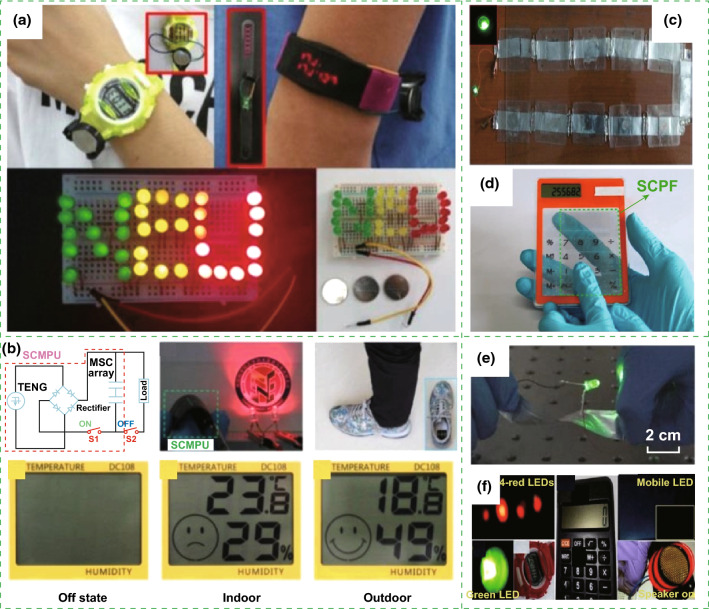
**a** The all-solid-state SCPC can power smartwatch, sports bracelet, and LEDs. Reprinted with permission from Ref. [11]. **b** Circuit diagram of the energy supply mode and photographs of showing two LEDs being powered by the SCMPU, the SCMPU inserted in the insole of a shoe and using the SCMPU to drive a commercial hygrothermograph. Reprinted with permission from Ref. [15]. **c** Operation of green light-emitting diode using serially connected SCSPCs as the power source. Reprinted with permission from Ref. [17]. **d** Photograph of a calculator driven by the SCPF. Reprinted with permission from Ref. [18]. **e** Photographs of the twisted Li-ion battery to light up a green LED. Reprinted with permission from Ref. [19]. **f** Photographs of the fabricated devices can instantaneous lightning of four commercial red LEDs upon repeated finger imparting and lightning of a green LED light with adequate intensity. Meanwhile, power-up small electronic wrist watch, commercial digital calculator, mobile LCD screen, and portable speaker. Reprinted with permission from Ref. [21]

**Fig. 14:**
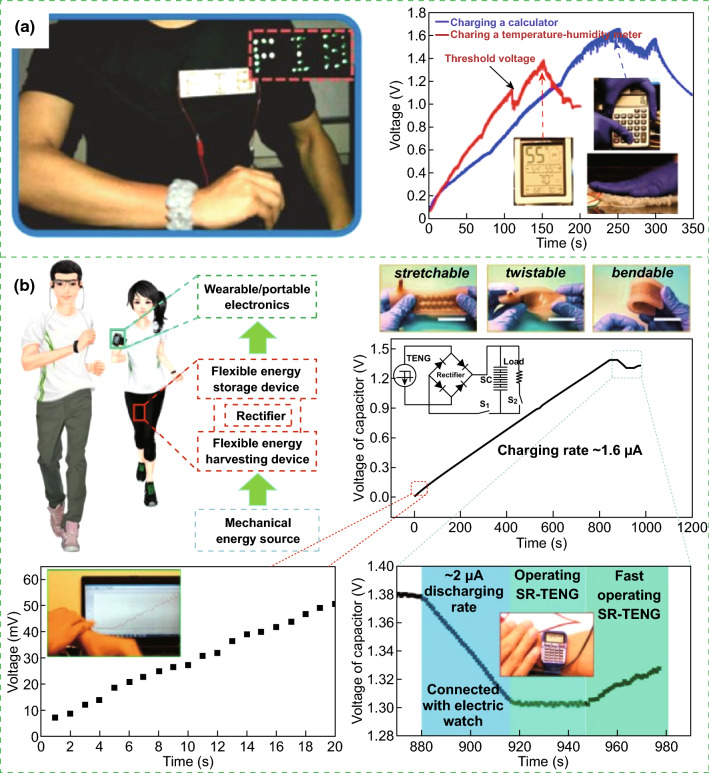
**a** LED warning sign on a shirt, that is, “FIB,” is lighted up by tapping a TENG bracelet wrapped on the wrist. Charging curves of two-series SCs by manually tapping the TENG fabric. The inset pictures are the temperature–humidity meter (left), calculator (upper right), and hand tapping fabric (lower right). Reprinted with permission from Ref. [22]. **b** Application of the all-in-one shape-adaptive self-charging power package in conventional wearable electronics. Reprinted with permission from Ref. [26]

**Fig. 15:**
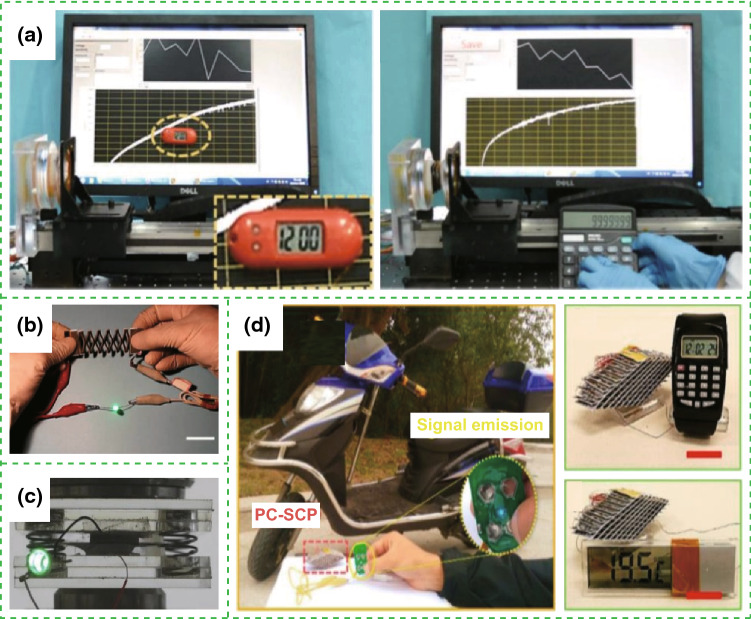
**a** Photograph of the self-charging power system powering an electronic watch and a calculator at 5 Hz. Reprinted with permission from Ref. [24]. **b** Photograph of the integrated KP-SC (6 units, 3 devices in series) lighting up a single commercial green LED under cycling stretching movement. Reprinted with permission from Ref. [26]. **c** Photograph of a green LED that can be lighted up by the convoluted power device as an SLB. Reprinted with permission from Ref. [27]. **d** Demonstration of a self-powered wireless remote control by incorporating a PC-SCPU, digital electric watch and temperature meter driven by manually tapping the PC-SCPU. Reprinted with permission from Ref. [28]

The fabricated nanogenerator-based self-charging energy storage devices can be utilized as a power source for powering certain electric devices. The all-solid-state SCPC can power smartwatch, sports bracelet, and LEDs, as illustrated in Fig. 13a [11]. Figure 13b presents that two lighted LEDs can be powered by the self-charging miro-supercapacitor power unit (SCMPU), where the SCMPU was inserted in the insole of a shoe to drive a commercial hygrothermograph [15]. Operation of green light-emitting diode uses integrated SCSPCs in series as the power source, as displayed in Fig. 13c [17]. Figure 13d illustrates that a calculator can be driven by the SCPF [18]. Figure 13e presents that the twisted Li-ion battery can be used to light up a green LED [19]. Moreover, it can be used as a power supply for small electronic wrist watch, commercial digital calculator, mobile LCD screen, and portable speaker (Fig. 13f) [21]. As displayed in Fig. 14a, LED light signals of “FIB” can be lighted up by tapping a TENG bracelet wrapped around the wrist. Two SCs in series can be used to power the temperature–humidity meter and calculator [22]. Figure 15a displays a self-charging power system, which can be used to power an electronic watch and a calculator at 5 Hz [24]. The fabricated all-in-one shape-adaptive self-charging power package in conventional wearable electronics and the integrated KP-SC (6 units, 3 devices in series) light up a single commercial green LED under cycling stretching movement, as demonstrated in Figs. 14b and 15b, respectively [26]. Figure 15c displays a green LED that can be lighted up by the convoluted power device as an SLB [27], where a PC-SCPU-driven wireless remote control can be observed. Meanwhile, a PC-SCPU can be used as a power source for driving a digital electric watch and a temperature meter, as demonstrated in Fig. 15d [28]. Thus, these devices have a wide range of potential applications in electronic devices and wireless sensors.

## Summary and Outlook

This review is focused on the recent progress of nanogenerator-based self-charging energy storage devices. The major achievements in this field can be summarized as follows: (1) Various self-charging devices have been developed to scavenge the mechanical energy and store it in themselves, which can be used to power some small electronic devices. (2) Self-charging principles of the energy storage devices have been investigated in details, where piezoelectric and triboelectric effects have been used to explain the working mechanisms of these devices. (3) Substantial practical applications of self-charging energy storage devices have been demonstrated, ranging from wearable electronics, sports monitoring, wireless sensors, to daily electronics.

Although significant improvements have been achieved, some problems regarding the investigations of nanogenerator-based self-charging energy storage devices are needed to be addressed: (1) Device life is a very critical matter in practical application. More attention needs to be focused on the device life to realize long-life devices. It may be necessary to start with material selection, structure design, etc. (2) LIBs or supercapacitors can be used for self-charging energy storage devices; the capacities and impedances of them should match the output of the energy-harvesting systems for higher conversion efficiency. Moreover, cost, safety, and easiness to integrate these devices are also one of the prior concerns. (3) The development of self-charging energy storage devices in future should follow the trend of miniaturization, diversification, integration, and portability.

In summary, developing nanogenerator-based self-charging devices is one of the effective methods to solve issues of continuous energy supply of the next-generation microelectronic devices. A series of research results regarding scavenging and storing the mechanical energy has been obtained, but there are still several problems to be solved such as other energy scavenging and storage systems, device life. Owing to the unremitting efforts by a large number of researchers around the world, we believe that the self-charging storage devices will be extensively applied in our daily life in the near future, especially for wearable electronic devices and self-powered systems.

## References

[1] Tarascon JM, Armand M (2001). Issues and challenges facing rechargeable lithium batteries. Nature.

[2] Goodenough JB, Park K (2013). The Li-ion rechargeable battery: a perspective. J. Am. Chem. Soc..

[3] Zhu Y, Murali S, Stoller MD, Ganesh KJ, Cai W (2011). Carbon-based supercapacitors produced by activation of grapheme. Science.

[4] Wang ZL, Song JH (2006). Piezoelectric nanogenerators based on zinc oxide nanowire arrays. Science.

[5] Fan FR, Tian ZQ, Wang ZL (2012). Flexible triboelectric generator. Nano Energy.

[6] Zhao K, Wang ZL, Yang Y (2016). Self-powered wireless smart sensor node enabled by an ultrastable, highly efficient, and superhydrophobic-surface-based triboelectric nanogenerator. ACS Nano.

[7] Diaz-Gonzalez F, Sumper A, Gomis-Bellmunt O (2012). A review of energy storage technologies for wind power applications. Renew. Sustain. Energy Rev..

[8] Wang ZL, Jiang T, Xu L (2017). Toward the blue energy dream by triboelectric nanogenerator networks. Nano Energy.

[9] Wang Y, Yang Y (2019). Superhydrophobic surfaces-based redox-induced electricity from water droplets for self-powered wearable electronics. Nano Energy.

[10] Quan T, Wang X, Wang ZL, Yang Y (2015). Hybridized electromagnetic–triboelectric nanogenerator for a self-powered electronic watch. ACS Nano.

[11] He H, Fu Y, Zhao T, Gao X, Xing L, Zhang Y, Xue X (2017). All-solid-state flexible self-charging power cell basing on piezo-electrolyte for harvesting/storing body-motion energy and powering wearable electronics. Nano Energy.

[12] Zhang Y, Zhang Y, Xue X, Cui C, He B, Nie Y, Deng P, Wang ZL (2014). PVDF–PZT nanocomposite film based self-charging power cell. Nanotechnology.

[13] Kim YS, Xie Y, Wen X, Wang S, Kim SJ, Song HK, Wang ZL (2015). Highly porous piezoelectric PVDF membrane as effective lithium ion transfer channels for enhanced self-charging power cell. Nano Energy.

[14] Pankratov D, Falkman P, Blum Z, Shleev S (2014). A hybrid electric power device for simultaneous generation and storage of electric energy. Energy Environ. Sci..

[15] Luo J, Fan FR, Jiang T, Wang Z, Tang W, Zhang C, Liu M, Cao G, Wang ZL (2015). Integration of micro-supercapacitors with triboelectric nanogenerators for a flexible self-charging power unit. Nano Res..

[16] Xing L, Nie Y, Xue X, Zhang Y (2014). PVDF mesoporous nanostructures as the piezo-separator for a self-charging power cell. Nano Energy.

[17] Ramadoss A, Saravanakumar B, Lee SW, Kim YS, Kim SJ, Wang ZL (2015). Piezoelectric-driven self-charging supercapacitor power cell. ACS Nano.

[18] Luo J, Tang W, Fan FR, Liu C, Pang Y, Cao G, Wang ZL (2016). Transparent and flexible self-charging power film and its application in a sliding unlock system in touchpad technology. ACS Nano.

[19] Zhao K, Yang Y, Liu X, Wang ZL (2017). Triboelectrifcation-enabled self-charging lithium-ion batteries. Adv. Energy Mater..

[20] Zhao J, Li H, Li C, Zhang Q, Sun J (2018). MOF for template-directed growth of well-oriented nanowire hybrid arrays on carbon nanotube fibers for wearable electronics integrated with triboelectric nanogenerators. Nano Energy.

[21] Maitra A, Karan SK, Paria S, Das AK, Bera R (2017). Fast charging self-powered wearable and flexible asymmetric supercapacitor power cell with fish swim bladder as an efficient natural bio-piezoelectric separator. Nano Energy.

[22] Dong K, Wang YC, Deng J, Dai Y, Zhang SL (2017). A highly stretchable and washable all-yarn based self-charging knitting power textile composed of fiber triboelectric nanogenerators and supercapacitors. ACS Nano.

[23] Wen Z, Yeh MH, Guo H, Wang J, Zi Y (2016). Self-powered textile for wearable electronics by hybridizing fiber-shaped nanogenerators, solar cells, and supercapacitors. Sci. Adv..

[24] Sun N, Wen Z, Zhao F, Yang Y, Shao H (2017). All flexible electrospun papers based self-charging power system. Nano Energy.

[25] Gao T, Zhao K, Liu X, Yang Y (2017). Implanting a solid Li-ion battery into a triboelectric nanogenerator for simultaneously scavenging and storing wind energy. Nano Energy.

[26] Guo H, Yeh MH, Lai YC, Zi Y, Wu C, Wen Z, Hu C, Wang ZL (2016). All-in-one shape-adaptive self-charging power package for wearable electronics. ACS Nano.

[27] Liu X, Zhao K, Wang ZL, Yang Y (2017). Unity convoluted design of solid Li-ion battery and triboelectric nanogenerator for self-powered wearable electronics. Adv. Energy Mater..

[28] Guo H, Yeh MH, Zi Y, Wen Z, Chen J, Liu G, Hu C, Wang ZL (2017). Ultralight cut-paper-based self-charging power unit for self-powered portable electronic and medical systems. ACS Nano.

[29] Pu X, Hu W, Wang ZL (2018). Toward wearable self-charging power systems: the integration of energy-harvesting and storage devices. Small.

[30] Pu X, Li L, Song H, Du C, Zhao Z, Jiang C, Cao G, Hu W, Wang ZL (2015). A self-charging power unit by integration of a textile triboelectric nanogenerator and a flexible lithium-ion battery for wearable electronics. Adv. Mater..

[31] Pu X, Li L, Liu M, Jiang C, Du C, Zhao Z, Hu W, Wang ZL (2016). Wearable self-charging power textile based on flexible yarn supercapacitors and fabric nanogenerators. Adv. Mater..

[32] Liu M, Cong Z, Pu X, Guo W, Liu T, Li M, Zhang Y, Hu W, Wang ZL (2019). High-energy asymmetric supercapacitor yarns for self-charging power textiles. Adv. Funct. Mater..

[33] Chen J, Guo H, Pu X, Wang X, Xi Y, Hu C (2018). Traditional weaving craft for one-piece self-charging power textile for wearable electronics. Nano Energy.

[34] Song Y, Wang H, Cheng X, Li G, Chen X, Chen H, Miao L, Zhang X, Zhang H (2019). Traditional weaving craft for one-piece self-charging power textile for wearable electronics. Nano Energy.

[35] Pu X, Liu M, Li L, Zhang C, Pang Y, Jiang C, Shao L, Hu W, Wang ZL (2016). Efficient charging of li-ion batteries with pulsed output current of triboelectric nanogenerators. Adv. Sci..

[36] Wei G, Wang Z, Zhu R, Kimura H (2018). PVDF/BCT–BZT nanocomposite film for a piezo-driven self-charging power cell batteries and energy storage. J. Electrochem. Soc..

[37] Pazhamalai P, Krishnamoorthy K, Mariappan VK, Sahoo S, Manoharan S, Kim S-J (2018). A high efficacy self-charging MoSe_2_ solid-state supercapacitor using electrospun nanofibrous piezoelectric separator with ionogel electrolyte. Adv. Mater. Interfaces.

[38] Yuan L, Xiao X, Ding T, Zhong J, Zhang X (2012). Paper-based supercapacitors for self-powered nanosystems. Angew. Chem. Int. Ed..

